# Diseases of the Colon

**Published:** 1904-11-26

**Authors:** 


### DISEASES OF THE COLON.

**Ulcerative Affections.**—These may be grouped into six main classes, according to Allchin:[1] (1) Ulceration resulting from severe catarrhal inflammation, with which may be classed stercoral ulcers and those observed in mercurial and lead poisoning. (2) Ulceration from vascular disturbance and lardaceous disease. (3) Certain specific ulcerations, such as typhoid, tubercle, mycosis, anthrax, and dysentery. (4) New growths. (5) Ulceration from without. (6) Trophic ulceration associated with central nervous lesions. Besides these are two special ulcerative conditions—follicular ulceration limited to the colon, chronic but rarely fatal, and "ulcerative colitis" steadily progressing to a fatal termination and frequently, but not constantly, associated with chronic Bright's disease. This condition resembles tropical dysentery and may be microbic in origin, but is of less acute character, being without the tenesmus, pain, and watery stools. The ulceration, however, is more general and the fatal termination much more certain. "Asylum dysentery" Allchin regards as intermediate between true dysentery and ulcerative colitis, but Eyre[2] has shown as the result of bacteriological investigations of an epidemic at one of the London County asylums that a bacillus identical with Shiga's can be isolated from the stools in many cases, and that the blood of some patients suffering from asylum dysentery will agglutinate B. dysenteriæ isolated from tropical cases. In chronic cases, however, he found the isolation of the typical bacillus very difficult, owing to the number of other organisms (B. coli, etc.) present. Dysenteric stools have been described by Duncan[3] as occurring in 10 varieties. (1) In milder cases a solid motion covered with greyish mucus is followed by small quantities of offensive mucus with minute fæcal lumps. Small quantities of mucus stained with fæces or blood and occasional scybala are then passed. The prognosis here is good. (2) In more severe cases whitish or coloured jelly-like mucus follows the fæcal discharge. There is a considerable amount of blood and clots. The motions are frequent and of two or three drachms in quantity. (3) Purulent stools containing small masses of blood-clot and membrane. (4) Pure blood. (5) Pure pus. (6) Lumps of tough mucus resembling frog's spawn. (7) Very offensive pulpy stools without blood or mucus. (8) Fluid fæcal matter. (9) Very offensive blackish fluid containing gangrenous lumps. (10) Amœbic stools which are far more liquid, mucoid with purulent shreddy material in them, and occasionally green, pultaceous, or gangrenous. The prognosis in the last four forms is bad. In chronic dysentery the stools vary considerably; they are thin and watery, and may be very offensive. Mucus, blood, or pus may occur in various amounts. The prognosis here is generally thought unfavourable, but good results may be obtained by appropriate treatment.

**Mucomembranous Colitis.**—This condition is described by De Langenhagen[4] as a chronic catarrhal inflammation of the colon of a constitutional character, neither parasitic nor contagious, and occurring in gouty and neurotic subjects. It is characterised by pain, constipation, sometimes alternating with diarrhœa and generally preceding the passage of quantities of mucus mixed with shreddy or membranous material derived from the dried secretions. Intestinal sand may be passed. There is also general depression, associated with various reflex disturbances and sometimes pronounced neurasthenia. The treatment consists of dieting, irrigation, and doses of certain mineral waters. The diet consists in the exclusion of all green vegetables, highly seasoned and fatty foods, wines, spirits, and most raw fruits. Bland carbohydrate foods, milk (generally diluted), and plain lean meats may be given with pulses, finely mashed. Bread in small quantities and a little mashed potato are allowed. Enemas and laxatives should be given to keep the bowel evacuated, and in spite of some objections to the measure, intestinal lavage should be practised in nearly all cases. De Langenhagen considers the waters of Plombières and Châtelguyon have no rivals in England. The former he recommends for cases where constitutional symptoms predominate (nervous or arthritic) and the latter to combat simple constipation. As to drugs, hot applications to the abdomen and belladonna suppositories may be used to relieve pain, but otherwise hygienic measures alone are to be relied on for effecting a cure. In addition to diseases like mucous colitis, which is held to be causally related to definite nervous disorder, Allchin[5] points out that all diseases of the colon are peculiarly apt to be accompanied by mental or nervous symptoms. He points out that apart from mental symptoms associated with severe reflected pain of visceral origin, auto-intoxication and the gouty condition may play a part, as in mucous colitis, by direct poisoning of the nervous centres. He also notes the possibility that poisons generated in the intestine may act through the vaso-motor system, which is certainly the case in some instances where flushings and cutaneous rashes are produced.

**Atony and Dilatation.**—Apart from secondary dilatation following obstruction, Allchin[6] recognises this as a primary condition, often induced by neglect, overdrugging, and fevers, but sometimes "idiopathic." Acute, often fatal, cases occur. This condition illustrates the similarity between the colon and the stomach, which is further emphasised by the following facts :—Neither stomach nor colon can be considered as essential to digestion, though their functions are no doubt important. Both are much more liable to malignant disease than the small intestine, and are more frequently inflamed, ulcerated, and dilated. Mucous colitis may be regarded as the analogue of hyperchlorhydria.

**Syphonage in the Colon.**—It is maintained by Leftwich[7] that the contents of the colon are moved on mechanically, the longitudinal bands of muscle keeping the gut in a condition of rigidity and patency. In support of this view he points out that saline purges do not act well if taken before bedtime, but only when the erect position is maintained, and draws attention to the occasional occurrence of morning diarrhœa. He argues that in cases of stricture the recumbent position should be avoided, that in typhoid fever the patient should sit up, and ascribes the occurrence of intussusception to cæcal vacua. Keith,[8] on the other hand, notes that the contents of the large intestine are not suitable for syphonage, that the muscular coats of the colon are well developed, and that peristaltic movements may be seen in them. Moreover, he doubts if the colon can ever be at once patent and rigid—necessary conditions for a syphon.

**Constipation and Diarrhœa.**—In addition to muscular and nervous activity and regularity of habit, it seems that an adequate residue from the food is necessary to ensure the proper action of the colon. Lohrisch[9] finds that in chronic constipation the motions are not only less frequent but smaller than normal, and that this diminution in size is not only due to a diminution in the amount of water but in solid residue also. In these cases the total amount of calories excreted is diminished and the amount of nitrogen, fat, and carbohydrates, as well as cellulose in the motions is less. This he sets down to a too perfect absorption of food. In constipation due to opium, only the amount of water is reduced, and the amount of dry residue is larger than normal. The number of intestinal bacteria in the cases described is smaller than normal, so that the reduction in waste food-stuffs cannot be ascribed to increased bacterial activity. This, as has been shown by Tabora,[10] tends to produce diarrhœa, especially in cases of imperfect gastric digestion. If in persons suffering from achylia the proteid constituents of diet are increased, the ethereal sulphates and indican eliminated are also increased, owing to the greater amount of pabulum afforded to the bacteria which set up putrefactive changes in the intestine. The products of these putrefactive changes irritate the bowel and set up peristalsis. In the treatment of diarrhœa caused by bacterial or other fermentative processes in the intestine Frenkel[11] recommends the peroxide of magnesium (Mg. $O_2$) known as hopogan. It is non-poisonous and non-astringent and acts by virtue of the nascent oxygen given off in contact with putrescent matter. It may be given in doses of one gram (gr. 15) per diem and increased to three grams (gr. 40). It is said by some authorities to be useless in diarrhœa of serous, vaso-motor, or tuberculous origin, but others have observed good effects in the last condition. In diarrhœa caused by new growths it is not of any value, but in 22 cases of presumably fermentative diarrhœa it acted promptly in 20.

[1] Clin. Jour., July 20, 1904, p. 215. [2] Brit. Med. Jour., April 30, 1904, p. 1002. [3] Edin. Med. Jour., April, 1904, p. 349. [4] Lancet, April 30, 1904, p. 1186. [5] Loc. cit., p. 218. [6] Loc. cit., p 217. [7] Lancet, May 14, 1904, p. 1367. [8] Ibid. [9] Med. Rec., May 21, 1904, p. 818. [10] Ibid., June 11, 1904, p. 971. [11] Lancet, July 9, 1904, p. 82.

